# Supplementary material for: Abundant pleiotropy across neuroimaging modalities identified through a multivariate genome-wide association study
Source: Nat Commun. 2024 Mar 26;15:2655. doi: 10.1038/s41467-024-46817-4 (PMC10965919; doi:10.1038/s41467-024-46817-4)
Supplement: Supplementary file 3 — Description of Additional Supplementary Files [file 41467_2024_46817_MOESM3_ESM.pdf]

Description of Additional Supplementary Files

File Name: Supplementary Data 1
Description: SNP-based heritability estimates from LDSC per phenotype derived from either structural, functional or diffusion MRI

File Name: Supplementary Data 2
Description: Genome-wide significant loci identified through MOSTest in UK Biobank

File Name: Supplementary Data 3
Description: Replication rates of UKB lead SNPs in ABCD (European and mixed ancestry samples)

File Name: Supplementary Data 4
Description: Genome-wide significant genes identified through MAGMA in UKB

File Name: Supplementary Data 5
Description: Genome-wide significant loci identified through MOSTest in ABCD with European and mixed ancestries

File Name: Supplementary Data 6
Description: Genome-wide significant genes identified through MAGMA in ABCD

File Name: Supplementary Data 7
Description: Single-modality and cross-modality genome-wide significant loci identified through MOSTest in UK Biobank

File Name: Supplementary Data 8
Description: Modality-specific and cross-modality genome-wide significant genes identified through MAGMA in UK Biobank

File Name: Supplementary Data 9
Description: ANNOVAR enrichment estimates for lead SNPs coming from different parts of the Venn Diagram

File Name: Supplementary Data 10
Description: Significantly enriched gene-sets (Gene Ontology) based on modality-specific and cross-modality genes

File Name: Supplementary Data 11
Description: Cell-type specific enrichment analysis Bhaduri et al

File Name: Supplementary Data 12
Description: Sample characteristics for the cohorts used in different types of cFDR analyses

File Name: Supplementary Data 13
Description: Genome-wide significant loci identified through the original GWAS or conditional FDR

File Name: Supplementary Data 14
Description: Sing concordance of genome-wide significant loci in independent samples

File Name: Supplementary Data 15
Description: Positionally mapped genes from the original GWAS or conditional FDR loci

File Name: Supplementary Data 16
Description: Tissue enrichment analysis for condFDR and orignal genes

File Name: Supplementary Data 17
Description: Polygenic score results using original disorder GWAS summary statistics or conditional FDR summary statistics conditioned on multimodal MOSTest

File Name: Supplementary Data 18
Description: Sample characteristics for the discovery (UKB) and replication (ABCD) multivariate GWAS cohorts used in this study

File Name: Supplementary Data 19
Description: Regularization parameter settings for each run of MOSTest during discovery and replication
