# Supplementary material for: Abundant pleiotropy across neuroimaging modalities identified through a multivariate genome-wide association study
Source: Nat Commun. 2024 Mar 26;15:2655. doi: 10.1038/s41467-024-46817-4 (PMC10965919; doi:10.1038/s41467-024-46817-4)
Supplement: Supplementary file 5 — Reporting Summary [file 41467_2024_46817_MOESM5_ESM.pdf]

nature portfolio

Corresponding author(s): EP Tissink, PhD
OA Andreassen, MD PhD

Last updated by author(s): February 16, 2024

Reporting Summary

Nature Portfolio wishes to improve the reproducibility of the work that we publish. This form provides structure for consistency and transparency in reporting. For further information on Nature Portfolio policies, see our Editorial Policies and the Editorial Policy Checklist.

Statistics

For all statistical analyses, confirm that the following items are present in the figure legend, table legend, main text, or Methods section.

n/a	Confirmed	
☐	☒	The exact sample size (*n*) for each experimental group/condition, given as a discrete number and unit of measurement
☐	☒	A statement on whether measurements were taken from distinct samples or whether the same sample was measured repeatedly
☐	☒	The statistical test(s) used AND whether they are one- or two-sided<br>*Only common tests should be described solely by name; describe more complex techniques in the Methods section.*
☐	☒	A description of all covariates tested
☐	☒	A description of any assumptions or corrections, such as tests of normality and adjustment for multiple comparisons
☐	☒	A full description of the statistical parameters including central tendency (e.g. means) or other basic estimates (e.g. regression coefficient) AND variation (e.g. standard deviation) or associated estimates of uncertainty (e.g. confidence intervals)
☐	☒	For null hypothesis testing, the test statistic (e.g. *F*, *t*, *r*) with confidence intervals, effect sizes, degrees of freedom and *P* value noted<br>*Give P values as exact values whenever suitable.*
☒	☐	For Bayesian analysis, information on the choice of priors and Markov chain Monte Carlo settings
☒	☐	For hierarchical and complex designs, identification of the appropriate level for tests and full reporting of outcomes
☐	☒	Estimates of effect sizes (e.g. Cohen's *d*, Pearson's *r*), indicating how they were calculated

Our web collection on statistics for biologists contains articles on many of the points above.

Software and code

Policy information about availability of computer code

Data collection	This study used data collected by UK Biobank, no new data was collected by the authors of the paper. For a description of the procedure in the UK Biobank, see Bycroft et al., Nature (2018).
Data analysis	Structural phenotypes were derived from Freesurfer v5.3, available at https://surfer.nmr.mgh.harvard.edu<br>Functional phenotypes were derived using the FSLNets toolbox, code to do so is available at https://fsl.fmrib.ox.ac.uk/fsl/fslwiki/FSLNets<br>Diffusion phenotypes were derived using Matlab v.2017a, code to do so is available at https://github.com/cmig-research-group/RSIGWAS<br>Conditional FDR analysis was run using pleioFDR, available at https://github.com/precimed/pleiofdr<br>Genotypes were quality controlled and analysed using PLINK2, available at https://www.cog-genomics.org/plink/2.0/<br>MOSTest was used for multivariate analyses, available at https://github.com/precimed/mostest<br>Polygenic scores were analysed with PRSice2, available at https://choishingwan.github.io/PRSice/<br>Heritability and genetic correlation were estimated using LD Score Regression, available at https://github.com/bulik/ldsc<br>Functional Mapping and Annotation of Genome-Wide Association Studies (FUMA) including ANNOVAR, Gene Ontology, MAGMA is available at https://fuma.ctglab.nl<br>Code to obtain the results presented in this manuscript are available via https://github.com/EPTissink/MOSTest-multimodal.

For manuscripts utilizing custom algorithms or software that are central to the research but not yet described in published literature, software must be made available to editors and reviewers. We strongly encourage code deposition in a community repository (e.g. GitHub). See the Nature Portfolio guidelines for submitting code & software for further information.

Data

Policy information about availability of data

All manuscripts must include a data availability statement. This statement should provide the following information, where applicable:

- Accession codes, unique identifiers, or web links for publicly available datasets
- A description of any restrictions on data availability
- For clinical datasets or third party data, please ensure that the statement adheres to our policy

The genome-wide summary statistics generated in this study have been made publicly available via https://cncr.nl/research/summary_statistics/ and GWAS Catalog (accession codes GCST90319487-GCST90319490).
The individual-level data that support the discovery findings of this study are available from UK Biobank but restrictions apply to the availability of these data, which were used under license no. 27412 for the current study. All researchers who wish to access this resource must register with UK Biobank by completing the registration form in the Access Management System.
Data used in the preparation of this article were obtained from the Adolescent Brain Cognitive DevelopmentSM (ABCD) Study (https://abcdstudy.org), held in the NIMH Data Archive (NDA). ABCD data used for replication in this study is registered under the NDA study register at https://doi.org/10.15154/1527969.
Data from the Norwegian Mother, Father and Child Cohort Study and the Medical Birth Registry of Norway used in this study are managed by the national health register holders in Norway (Norwegian Institute of public health) and can be made available to researchers, provided approval from the Regional Committees for Medical and Health Research Ethics (REC), compliance with the EU General Data Protection Regulation (GDPR) and approval from the data owners.

Research involving human participants, their data, or biological material

Policy information about studies with human participants or human data. See also policy information about sex, gender (identity/presentation), and sexual orientation and race, ethnicity and racism.

Reporting on sex and gender	Biological sex was used as a covariate in the multivariate GWAS analyses in this study. No sex-stratified GWAS have been performed, due to the lack of sufficient sample size.
Reporting on race, ethnicity, or other socially relevant groupings	UKB samples: white British genetic ancestry. ABCD samples: first used after filtering on European ancestry, secondly without genetic ancestry filtering.
Population characteristics	UKB samples: 30,106/31,023/34,029 individuals have passed the quality controls (depending on the modality dMRI/fMRI/sMRI). Mean age is 64 years and 52% of them are female.<br>ABCD samples: 7,277/7,853/8,607 individuals passed quality control (depending on the modality fMRI/dMRI/sMRI). Mean age is 9.94 years old and 47% of them are female.
Recruitment	UKB is a cohort design, recruiting adults from the UK and having MRI scans from the three dedicated MRI scanners.<br>ABCD is a longitudinal cohort design, recruiting adolescents across 21 sites in the USA.
Ethics oversight	NIMH Data Archive and UKBiobank hold the oversight of data usage for this study.

Note that full information on the approval of the study protocol must also be provided in the manuscript.

Field-specific reporting

Please select the one below that is the best fit for your research. If you are not sure, read the appropriate sections before making your selection.

☒ Life sciences ☐ Behavioural & social sciences ☐ Ecological, evolutionary & environmental sciences

For a reference copy of the document with all sections, see nature.com/documents/nr-reporting-summary-flat.pdf

Life sciences study design

All studies must disclose on these points even when the disclosure is negative.

Sample size	No statistical methods were used to predetermine the sample size. The samples were selected based on genetic and MRI data availability, successfully passing MRI data quality control, and successfully passed genomic data quality control.
Data exclusions	Basic data quality controls were imposed as described in the Methods section. Imaging data of ABCD has to have quality score greater than 0.9 (0 - 1 quality rating). Genomic data has to have call rate greater than 95% and imputation quality score greater than 0.9.
Replication	No randomness was introduced in sample allocation. Analyses were controlled for age, age^2, sex, genetic principal components, genotype array, scanner, and modality specific covariates. The latter included Euler number, and total surface area, mean thickness or intracranial volume (sMRI), signal to noise ratio and motion (fMRI), and intracranial volume (dMRI).
Randomization	We designed the study into one discovery set (UKB) and two validation sets (ABCD EUR, ABCD mixed ancestry). The reproducibility is evaluated based on significance testing on validation sets given predetermined discovery SNPs, and converging evidence for genetic overlap (loci and genes). The genetic overlap finding was replicated. Locus replication rates were modest, but in line with previous literature.

Blinding	Investigators were not blinded to the sample allocation. The only groups defined in this analyses is whether individuals belonged to discovery set (UKB) or validation sets (ABCD), which researchers in this study needed to know in order to perform analyses.

Reporting for specific materials, systems and methods

We require information from authors about some types of materials, experimental systems and methods used in many studies. Here, indicate whether each material, system or method listed is relevant to your study. If you are not sure if a list item applies to your research, read the appropriate section before selecting a response.

Materials & experimental systems

n/a	Involved in the study
☒	☐ Antibodies
☒	☐ Eukaryotic cell lines
☒	☐ Palaeontology and archaeology
☒	☐ Animals and other organisms
☒	☐ Clinical data
☒	☐ Dual use research of concern
☒	☐ Plants

Methods

n/a	Involved in the study
☒	☐ ChIP-seq
☒	☐ Flow cytometry
☐	☒ MRI-based neuroimaging

Plants

Seed stocks	*Report on the source of all seed stocks or other plant material used. If applicable, state the seed stock centre and catalogue number. If plant specimens were collected from the field, describe the collection location, date and sampling procedures.*
Novel plant genotypes	*Describe the methods by which all novel plant genotypes were produced. This includes those generated by transgenic approaches, gene editing, chemical/radiation-based mutagenesis and hybridization. For transgenic lines, describe the transformation method, the number of independent lines analyzed and the generation upon which experiments were performed. For gene-edited lines, describe the editor used, the endogenous sequence targeted for editing, the targeting guide RNA sequence (if applicable) and how the editor was applied.*
Authentication	*Describe any authentication procedures for each seed stock used or novel genotype generated. Describe any experiments used to assess the effect of a mutation and, where applicable, how potential secondary effects (e.g. second site T-DNA insertions, mosiacism, off-target gene editing) were examined.*

Magnetic resonance imaging

Experimental design

Design type	This was an observational study without experimental design.
Design specifications	NA
Behavioral performance measures	NA

Acquisition

Imaging type(s)	Structural, Functional, Diffusion MRI
Field strength	3T
Sequence & imaging parameters	3D MPRAGE, sagittal, R=2, TI/TR=880/2000 ms, 1.0x1.0x1.0 mm, 208x256x256
Area of acquisition	Whole brain

Diffusion MRI ☒ Used ☐ Not used

Parameters	Mutli-shell diffusion data of UKB acquired with five b=0 s/mm2 frames and 100 non-collinear gradient directions, with 50 directions at b=1000 s/mm2 and 50 directions at b=2000 s/mm2.<br>Multi-shell diffusion MRI data of ABCD acquired with seven b=0 s/mm2 frames and 96 non-collinear gradient directions, with 6 directions at b=500 s/mm2, 15 directions at b=1000 s/mm2, 15 directions at b=200 s/mm2, and 60 directions at b=3000 s/mm2.

Preprocessing

Preprocessing software	UKB and ABCD structural and functional scans were preprocessed by the UKB and ABCD team. We proceeded with:<br>Structural: We employed a centralized and harmonized processing protocol including automated surface-based

morphometry and subcortical segmentation using Freesurfer v5.3 (recon-all).
Functional: computing functional brain networks using the FSLNets toolbox
Diffusion: ABCD processing pipeline, implemented in Matlab v.2017a.

Normalization

Intensity normalization, rigid body registration, and non-linear multi-channel registrations were performed to ensure the compatibility in the voxel level.

Normalization template

Structural: fsaverage
Diffusion: The common atlas used for registration is in RAS, while the study specific group average were obtained iteratively.

Noise and artifact removal

Processed through forward-reverse gradient warping, eddy current correction, and motion correction to reduce the spatial distortion and signal heterogeneities driven by scanner differences.

Volume censoring

No censoring

Statistical modeling & inference

Model type and settings

Multivariate GWAS

Effect(s) tested

Effect of each SNP, across the genome, on sets of neuroimaging derived phenotypes

Specify type of analysis: ☐ Whole brain ☒ ROI-based ☐ Both

Anatomical location(s)

Desikan-Killiany Atlas for structural MRI
Schaefer Atlas for functional MRI
Weighted sum test with principal component analysis for diffusion MRI

Statistic type for inference
(See Eklund et al. 2016)

Permutation-based

Correction

Bonferroni correction (p<5E-8/3)

Models & analysis

n/a	Involved in the study
☐	☒ Functional and/or effective connectivity
☒	☐ Graph analysis
☐	☒ Multivariate modeling or predictive analysis

Functional and/or effective connectivity

Pearson correlation

Multivariate modeling and predictive analysis

Imaging phenotypes were treated as multivariate dependent variables, while the independent variables include genetic variants and study specific covariates.
